# Combined effects of asbestos and cigarette smoke on the development of lung adenocarcinoma: Different carcinogens may cause different genomic changes

**DOI:** 10.3892/or.2014.3263

**Published:** 2014-06-13

**Authors:** KENTARO INAMURA, HIRONORI NINOMIYA, KIMIE NOMURA, EIJU TSUCHIYA, YUKITOSHI SATOH, SAKAE OKUMURA, KEN NAKAGAWA, AYAKO TAKATA, NORIHIKO KOHYAMA, YUICHI ISHIKAWA

**Affiliations:** 1 Division of Pathology, The Cancer Institute, Japanese Foundation for Cancer Research (JFCR), Koto-ku, Tokyo 135-8550, Japan; 2 Department of Thoracic Surgery, Kitasato University School of Medicine, Sagamihara, Kanagawa 228-8555, Japan; 3 Department of Thoracic Surgery, The Cancer Institute Hospital, JFCR, Koto-ku, Tokyo 135-8550, Japan; 4 Department of Preventive Medicine, St. Marianna University School of Medicine, Miyamae-ku, Kawasaki, Kanagawa 216-8511, Japan; 5 Faculty of Economics, Toyo University, Bunkyo-ku, Tokyo 12-8608, Japan

**Keywords:** lung cancer, asbestos, smoking, loss of heterozygosity, *p53* mutation

## Abstract

The carcinogens in cigarette smoke are distinct from asbestos. However, an understanding of their differential effects on lung adenocarcinoma development remains elusive. We investigated loss of heterozygosity (LOH) and the *p53* mutation in 132 lung adenocarcinomas, for which asbestos body burden (AB; in numbers per gram of dry lung) was measured using adjacent normal lung. All cases were classified into 9 groups based on a matrix of cumulative smoking (CS in pack-years; CS=0, 0<CS<25, ≥25 CS) and AB (AB=0, 0<AB<1,000, ≥1,000 AB). AB=0 indicates a lower level than the detection limit of ~100. LOH frequency increased only slightly with the elevation of CS in the AB=0 groups. In the AB>0 groups, LOH frequency increased as AB and/or CS was elevated and was significantly higher in the ≥1,000 AB, ≥25 CS group (p=0.032). *p53* mutation frequency was the lowest in the AB=0, CS=0 group, increased as AB and/or CS rose, and was significantly higher in the ≥1,000 AB, ≥25 CS group (p=0.039). *p53* mutations characteristic of smoking were frequently observed in the CS>0 groups contrary to non-specific mutations in the CS=0, AB>0 groups. Combined effects of asbestos and smoking were suggested by LOH and *p53* analyses. Sole exposure to asbestos did not increase LOH frequency but increased non-specific *p53* mutations. These findings indicate that the major carcinogenic mechanism of asbestos may be tumor promotion, acting in an additive or synergistic manner, contributing to the genotoxic effect of smoking. Since this study was based on a general cancer center’s experience, the limited sample size did not permit the consideration that the result was conclusive. Further investigation with a large sample size is needed to establish the mechanism of asbestos-induced lung carcinogenesis.

## Introduction

Lung cancer is one of the leading causes of cancer-related death in both men and women worldwide, and adenocarcinoma is the most predominant histologic subtype in many parts of the world. Tobacco smoke is clearly the most important factor associated with the development of lung cancer, accounting for 80–90% of all cases. Asbestos is another significant inhaled carcinogen, contributing to the development of ~5–7% of all lung cancers (1). Many studies on asbestos-related lung carcinogenesis have analyzed the genotoxic effects of asbestos; asbestos fibers induce DNA damage, chromosome aberrations, mitotic disturbances and gene mutations (2). In addition, asbestos fibers can stimulate a range of other effects including cell proliferation, chronic inflammation, enhanced gene expression, such as c-fos and c-jun overexpression, and transformation (3,4). Despite these studies, the efficacy of asbestos-exposure as a complete lung carcinogen, independent of tobacco smoke, has not been demonstrated in humans, since lung cancers of asbestos-exposed individuals frequently occur in smokers and ex-smokers. The majority of asbestos-related lung cancers may result from the combined effects of asbestos and carcinogens in tobacco smoke, with the possibility of a synergistic relationship first proposed by Doll (5). Hence, the mechanism of asbestos-induced lung carcinogenesis still remains unclear.

Both loss of heterozygosity (LOH) and the *p53* mutation are genetic alterations. LOH is frequently noted in cancer cells and is thought to occur through genetic instability at the chromosomal level. On the other hand, the *p53* mutation is a genetic alteration at the nucleotide level. Mutation in the *p53* tumor suppressor gene is the most frequently observed gene mutation in cancers. As described below, not only *p53* mutations but also LOH spectra differ in different cancer types associated with different etiologies. Previously we compared the frequency of LOH on all autosomal chromosomes among non-small cell lung carcinomas (6,7) as well as *p53* mutation patterns with adenocarcinoma cell morphology (8). The frequency of allelic loss on many chromosomal arms was commonly higher in squamous cell carcinomas than in adenocarcinomas. This result suggested that more cumulative genetic changes are associated with tumorigenesis in squamous cell carcinomas than contribute to adenocarcinomas, a pattern which may reflect a difference in the carcinogenic mechanisms responsible for the two histologies. In addition, we observed high frequencies of allelic losses on chromosomes 9p, 9q and 13q in squamous cell carcinomas, the majority of which were from smokers, and higher frequencies of allelic losses on these arms in adenocarcinomas from smokers than those from non-smokers. This loss of specific chromosomes associated with a particular histology is an example of LOH spectra reflecting etiology. The *p53* mutational spectra differ among cancers of various organs, and its frequency and mutational spectra can be said to reflect carcinogenic patterns characteristic of exogenous or endogenous factors and thus may be helpful for identification of the responsible agents, including, among others, cigarette smoke, aflatoxin B1 and ultraviolet light. Hence, the analysis of *p53* mutation can provide clues to the etiology of diverse tumors and to the function of specific regions of *p53* (9,10). The mutation pattern in smokers shows an excess of G:C to T:A transversions (34.2%), which are relatively uncommon in non-smokers or passive-smokers (16.6%) (11). These transversions often occur at codons 157, 158, 245, 248 and 273, experimentally identified as sites of adduct formation by benzo(a)pyrene, a single polycyclic aromatic hydrocarbon (PAH)-compound found in cigarette smoke. Other PAH-compounds also have a similar preference for adduct formation in these *p53* codons (12,13).

In the present study, to elucidate the combined effects of asbestos-exposure and smoking on development of lung adenocarcinomas, we used 132 lung adenocarcinomas, for which we already obtained all detailed smoking histories, comprehensive LOH data for all autosomal chromosomes (7), and *p53* mutation data.

## Materials and methods

### Patients and sample preparation

A total of 335 cases of lung adenocarcinoma were surgically removed at the Cancer Institute Hospital (CIH), Tokyo, Japan, between September 1989 and August 1996. Among the cases, fresh tumor tissues and corresponding normal lung and detailed smoking histories were successfully collected from 132 patients, which were used as materials in this study. Hence, they were collected semi-randomly without respect to asbestos-exposure status, and therefore provided a representative population for a cancer center in Japan. The clinicopathological data for these samples are summarized in Table I. We used a differentiation grading that was basically according to the former version of the Japanese Lung Cancer Society (14), as previously performed (15). Smoking history was surveyed intensively from patients and their families and presented as cumulative smoking (CS) in pack-years. The study protocol was approved by IRB of CIH and informed consent was obtained from all patients.

### Measurement of asbestos-exposure

Asbestos-body burden (AB; in numbers per gram of dry lung tissue) was measured using paraffin blocks of corresponding normal lung tissues by a polarizing microscope (16). The detection limit, which means no AB was found on the measuring filter sample, was ~100 AB/g (dry lung) and expressed as 0 in this study.

### A matrix of smoking-exposure and asbestos-exposure

To examine the dose-effect relationship of asbestos-exposure (presented as AB) and smoking-exposure (presented as CS in pack-years) on lung adenocarcinomas, we classified all cases into 9 groups based on a matrix of CS in pack-years: CS=0 (n=54, 41%), 0<CS<25 (n=18, 14%), ≥25 CS (n=60, 45%), and AB: AB=0 (n=64, 48%), 0<AB<1,000 (n=28, 21%), ≥1,000 AB (n=40, 31%). Since the patients were selected consecutively from surgical tumor files in a general cancer center, only 4 cases (3.0%) exceeded 5,000 in AB. To investigate the mechanism of asbestos-induced lung carcinogenesis in a representative population for a cancer center, not a biased population heavily exposed to asbestos, we divided the cases between AB <1,000 and ≥1,000 AB.

### LOH analysis

For LOH analysis, we performed Southern blotting. Experimental procedures and probes used were essentially the same as previously described (6,7). To facilitate the comparison, we used a fractional allelic loss (FAL) value, defined as: (number of chromosome arms with LOH)/(number of informative arms) for each case. Of 132 patients with adenocarcinomas, LOH data were available for 114 patients.

### p53 mutation analysis

Analysis of *p53* mutation was performed essentially as described elsewhere (8). Genomic DNA from fresh tumor samples was prepared and exons 4–8 and 10 of *p53* were analyzed by polymerase chain reaction and DNA sequencing. Of the 132 patients with adenocarcinomas, *p53* mutation data were available for 123 patients.

### Statistical analysis

For statistical analysis, we used the t-test, Fisher’s exact test, and Chi-square test, as appropriate. The two-sided significant level was set at p<0.05. Data were analyzed with the statistical software Stata version 11 (StataCorp., College Station, TX, USA).

## Results

**Figure 1:**
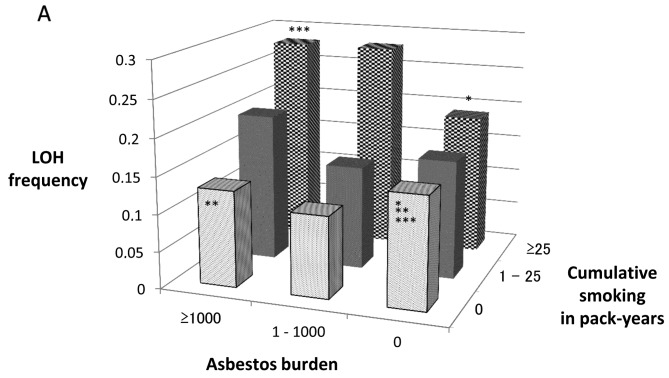

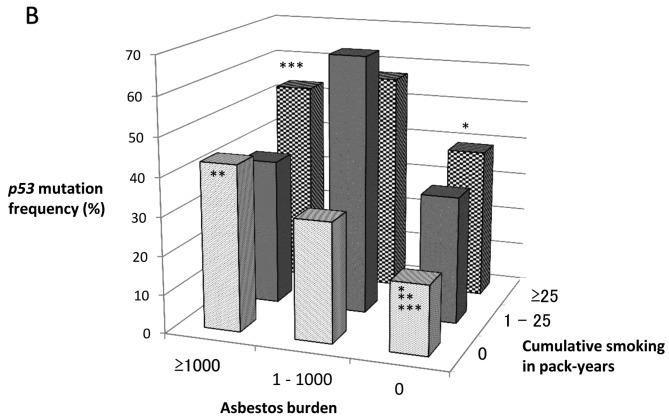
(A) Frequency of the loss of heterozygosity (LOH) in lung adenocarcinomas classified by cumulative smoking (CS) in pack-years (CS=0, 0<CS<25, ≥25 CS) and asbestos burden (AB) (AB=0, 0<AB<1,000, ≥1,000 AB). FAL, fractional allelic loss. ^*^p=0.30 (AB=0, CS=0 vs. AB=0, 25≤CS); ^**^p=0.69 (AB=0, CS=0 vs. ≥1,000 AB, CS=0); ^***^p=0.032 (AB=0, CS=0 vs. ≥1,000 AB, ≥25 CS). (B) p53 mutation frequency in lung adenocarcinomas classified by CS in pack-years (CS=0, 0<CS<25, 25≤CS) and AB (AB=0, 0<AB<1,000, 1,000≤AB). ^*^p=0.14 (AB=0, CS=0 vs. AB=0, 25≤CS); ^**^p=0.14 (AB=0, CS=0 vs. ≥1,000 AB, CS=0); ^***^p=0.039 (AB=0, CS=0 vs. ≥1,000 AB, ≥25 CS).

LOH frequency of lung adenocarcinomas classified by CS and AB is shown in Table II and Fig. 1A. LOH frequency increased only slightly correlating with the elevation of CS in the AB=0 groups, whereas, in the AB>0 groups, it increased as AB and/or CS was elevated and was significantly higher in the ≥1,000 AB, ≥25 CS group than in the AB=CS=0 group (p=0.032).

**Figure 2:**
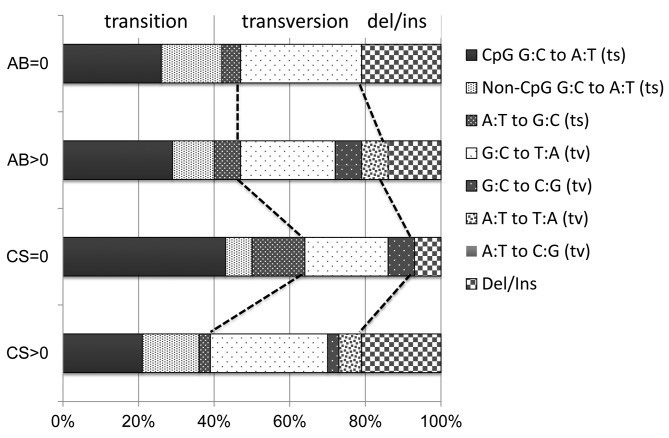
p53 mutation spectra in lung adenocarcinomas. AB=0 (n=19): lung adenocarcinomas from patients without AB (0<CS, n=15; CS=0, n=4). AB>0 (n=28): lung adenocarcinomas from patients with AB (0<CS, n=18; CS=0, n=10). CS=0 (n=14): lung adenocarcinomas from non-smokers (0<AB, n=10; AB=0, n=4). CS>0 (n=33): lung adenocarcinomas from smokers and ex-smokers (0<AB, n=18; AB=0, n=15). CS, cumulative smoking in pack-years; AB, asbestos burden; ts, transition; tv, transversion; Del/Ins, deletion/insertion.

Details of cases with *p53* mutations in lung adenocarcinomas are shown in Table III and summarized in Table IV. The *p53* mutation rates of pathological stage I and II–IV lung adenocarcinomas were 32% (18 of 57) and 44% (29 of 66), respectively, not significantly different by Fisher’s exact test (p=0.19). *p53* mutation frequency of lung adenocarcinomas classified by CS and AB are depicted in Fig. 1B. *p53* mutation frequency was the lowest in the AB=CS=0 group (18%), increased as AB and/or CS rose, and was significantly higher in the ≥1,000 AB, ≥25 CS group (53%) than in the AB=CS=0 group (p=0.039). Tobacco smoke, one of the most significant exogenous carcinogenic agents has been shown to frequently cause specific *p53* mutations, especially G:C to T:A transversion (17) at specific codons described as ‘hotspots’, such as codon 157, 158, 245, 248 and 273 (13). *p53* mutations characteristic of smoking, such as G:C to T:A transversion at the tobacco-specific codons were frequently observed in the CS>0 groups, whereas non-specific mutations were often detected in the CS=0, AB>0 groups (Tables III and IV). In the ≥1,000 AB, CS=0 group, there was only one transversion and no tobacco-specific codons for the six *p53* mutations. In contrast, in the AB=0, ≥25 CS group, there were five G:C to T:A transversions and five tobacco-specific codons among 13 *p53* mutations. Fig. 2 shows *p53* mutation spectra in lung adenocarcinomas, classified as smokers (A, n=33) or non-smokers (B, n=14) and asbestos-exposed (C, n=28) or not (D, n=19). Although *p53* mutation spectra varied depending on the status of smoking history, they showed little difference between asbestos-exposed or non-exposed. Whereas smokers had frequent G:C to T:A transversions, which are smoking-associated *p53* mutations, non-smokers had frequent G:C to A:T transitions at CpG sites associated with spontaneous mutations, consistent with previous reports (9,17).

**Figure 3:**
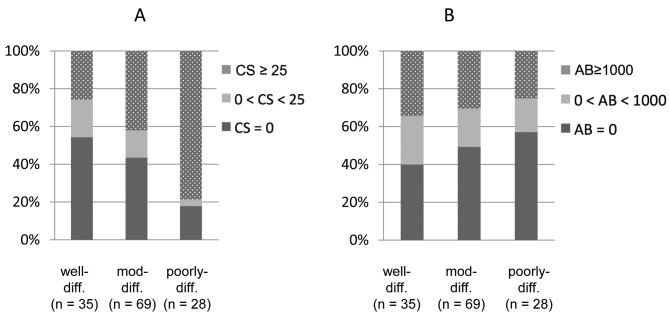
Cumulative smoking (CS) in pack-years (A) and asbestos burden (AB) (B) with reference to the histological differentiation grade. Although there was a significant relationship between CS and the differentiation grade (p=0.0010, Chi-square test), there was no correlation between AB and the differentiation grade (p=0.75). Well-diff., well-differentiated; mod-diff., moderately differentiated; poorly-diff., poorly differentiated.

With respect to tumor differentiation grade, a heavier smoking habit was associated with less-differentiated adenocarcinomas (Fig. 3A, p=0.0010, Chi-square test), in line with a previous study (18). On the other hand, there was no correlation between asbestos deposition and the differentiation grade (Fig. 3B, p=0.75).

## Discussion

Both tobacco smoke and asbestos fibers are significant inhaled carcinogens which contribute significantly to lung adenocarcinoma development. We previously revealed that chromosome instability and LOH, rather than minisatellite and microsatellite instability, play major roles in the development of lung adenocarcinomas (19). The LOH and *p53* spectra provide clues concerning the etiology and nature of carcinogenesis. To elucidate the carcinogenic mechanisms of two different inhaled carcinogens, asbestos and cigarette smoke, we investigated LOH on all autosomal chromosomes and measured asbestos burden (AB; asbestos body per gram of dry lung tissue) using corresponding normal lung tissue and investigated *p53* mutation employing fresh tumor samples.

The *p53* mutational spectra may be helpful for identification of the origins of the mutations that give rise to human cancers. For example, aflatoxin B1-associated hepatocellular carcinomas frequently have the specific *p53* mutations: G:C to T:A transversions at the 3rd base of codon 249, AGG to AGT (Arg to Ser) (20). Another example of a clearly characteristic ‘finger-print’ mutation in *p53* is the CC to TT double mutation in skin cancer (21). Exposure to UV light, a physical mutagen, produces distinctive pyrimidine dimers that, if unrepaired, can produce tandem mutations, most characteristically CC to TT transitions. Similar to these, the *p53* mutational spectra can provide clues to the etiology of cancers.

The possible role of asbestos-exposure in the genesis of *p53* mutations in lung cancers is less well understood. Husgafvel-Pursiainen *et al* investigated *p53* mutation of 105 lung cancers from smokers, comprising 53 squamous cell carcinomas, 39 adenocarcinomas and other 13 carcinomas, focusing on the presence or absence of asbestos-exposure (22). They found *p53* mutations in 39% of asbestos-exposed patients with lung cancer while the percentage was 54% in patients not exposed to asbestos, indicating that the *p53* mutations were less common among the cases with occupational asbestos-exposure than in the non-exposed cases. These results have not been verified yet by another study, and need additional examinations of smoking status.

In adenocarcinoma without asbestos-exposure or smoking-exposure, the *p53* mutation rate was the lowest. It increased in correlation with the elevation of asbestos-exposure and/or smoking-exposure. Adenocarcinomas associated with frequent smoking have characteristic *p53* mutations, especially G:C to T:A transversions (17), at specific ‘hotspot’ codons (13). However, adenocarcinomas associated only with asbestos-exposure had non-specific *p53* mutations, such as transitions which are thought to be caused by endogenous mechanisms associated with spontaneous events (9,17). Asbestos may work in a promoter-like manner. Production of reactive oxygen species and/or induction of tissue regeneration may be relevant.

Adenocarcinomas have different etiologies from squamous cell carcinomas, which can be reflected also in terms of LOH. As we revealed, LOH frequency was higher in squamous cell carcinomas than in adenocarcinomas (6,7). Poorly differentiated adenocarcinomas, which are often noted in smokers such as squamous cell carcinomas, have higher LOH frequency than differentiated adenocarcinomas, which have a relatively weaker association with smoking (23). Smoking induces complicated genetic changes in lung cancers.

One of the most intriguing recent discoveries in the field of lung cancer research is the identification of new driver mutations in lung adenocarcinomas, such as *EGFR* mutations (24,25) and *ALK* fusion (26). Both lung cancers with *EGFR* mutations or *ALK* translocations are characterized by negative or light smoking history. Lung cancers in non-smokers are considered to be less genetically complex than those in smokers and therefore they often have distinct characteristics developing on simple gene mutations for maintenance and survival. Consequently, patients with tumors harboring such simple oncogenic mutations represent good candidates who may stand to benefit from molecular-targeted drugs. To date, two-thirds of Japanese adenocarcinomas and a little more than half of Caucasian adenocarcinomas have mutually exclusive oncogenic mutations or other genetic alterations including *EGFR*, *KRAS*, *MET*, *ALK* and *HER2* (27). Asbestos-associated alterations in chromosomal regions, such as 19p13 (28), 9q33.1 (29) and 2p16 (30) have been identified. Whereas the smoking status has a significant association with driver mutations in lung adenocarcinomas, the relationship with asbestos-exposure remains unclear.

In adenocarcinomas without asbestos-exposure, the LOH frequency increased only slightly, correlating with the elevation in smoking-exposure. On the other hand, in adenocarcinomas with asbestos-exposure, the LOH frequency increased as asbestos-exposure and/or smoking-exposure was elevated. This suggests that asbestos-exposure in concert with smoking-exposure increases LOH frequency.

In the present study, lung adenocarcinomas, for which asbestos-exposure and smoking-exposure data could be obtained, were examined for LOH and the *p53* mutation. Combined effects of asbestos and cigarette smoke were suggested by these analyses. Asbestos-exposure alone did not increase the LOH frequency but increased non-specific *p53* mutations. These findings suggest that the major carcinogenic mechanism of asbestos in lung adenocarcinomas may be as a promoter, contributing to the genotoxic effect of cigarette smoke. Since this study was based on a general cancer center’s experience, the limited sample size does not permit consideration that the result is conclusive. Further investigation with a large sample size is required to establish the mechanism of asbestos-induced lung carcinogenesis.

## Figures and Tables

**Table I:** Clinicopathological data of the patients with lung adenocarcinomas analyzed in this study (n=132).

Clinicopathological features	No. of patients (%)
Age (years ± SD)	61±11
Gender	
Male	74 (56)
Female	58 (44)
Cumulative smoking	
CS=0	54 (41)
0<CS<25	18 (14)
≥25 CS	60 (45)
Asbestos burden	
AB=0	64 (48)
0<AB<1,000	28 (21)
≥1,000 AB <5,000	36 (27)
≥5,000 AB	4 (3)
pStage	
I	63 (48)
II–IV	69 (52)
Differentiation	
Well	35 (27)
Moderately	69 (52)
Poorly	28 (21)
Size (mm)	
<30	75 (57)
≥30	57 (43)

CS, cumulative smoking in pack-years; AB, asbestos burden; pStage, pathological stage. Percentages may not total 100, due to rounding.

**Table II:** FAL values (± SD) in lung adenocarcinomas, classified by AB and CS in pack-years.

	AB			
		
	0	1–1,000	≥1,000	Total
CS				
0	0.15 (±0.13) (n=20)	0.11 (±0.13) (n=16)	0.13 (±0.16) (n=13)	0.13 (±0.12) (n=49)
1–25	0.16 (±0.17) (n=4)	0.14 (±0.04) (n=3)	0.20 (±0.20) (n=7)	0.18 (±0.15) (n=14)
≥25	0.19 (±0.14) (n=28)	0.28 (±0.25) (n=6)	0.28 (±0.22) (n=17)	0.23 (±0.17) (n=51)
Total	0.17 (±0.12) (n=52)	0.15 (±0.16) (n=25)	0.21 (±0.18) (n=37)	0.18 (±0.15) (n=114)

FAL, fractional allelic loss; AB, asbestos burden; CS, cumulative smoking.

**Table III:** Details of the cases with *p53* mutations in lung adenocarcinomas.

Classified by CS and AB	Case no.	Gender	Age (years)	Diff.	Size (mm)	pStage	AB	CS	FAL	Mut type	Codon	Base change	Amino acid
CS=0	33	F	49	Mod	27	IIIB	0	0	0.13	ts	273	CGT to CAT	Asp→His
AB=0	70	F	26	W	43	IIIB	0	0	0.53	tv	176	TGC to TTC	Cys→Phe
	73	M	44	P	35	IIIA	0	0	0.35	ts	120	AAG to AGG	Lys→Arg
	74	F	70	W	20	IA	0	0	0.06	ts	248	CGG to CAG	Arg→Glu
CS=0	27	F	77	Mod	38	IIIB	187	0	0.18	tv	237	ATG to ATT	Met→Ile
0<AB<1,000	39	F	51	Mod	24	IIIB	214	0	0.05	ts	245	GGC to AGC	Gly→Ser
	47	F	65	W	24	IA	333	0	0.06	ts	335	CGT to CAT	Arg→His
	81	F	68	Mod	21	IA	671	0	0.05	tv	273	CGT to CTT	Asp→Leu
CS=0	3	F	51	Mod	33	IIIA	1,715	0	0.14	del	341	TTC to T---C	Frameshift
≥1,000 AB	7	F	72	Mod	60	IV	3,939	0	0.58	ts	138	GCC to GTC	Ala→Val
	14	F	57	Mod	40	IIIA	2,305	0	0.29	tv	138	GCC to CCC	Ala→Pro
	84	F	63	Mod	25	IIIB	1,000	0	0.21	ts	282	CGG to TGG	Arg→Trp
	113	F	67	Mod	32	IIIB	1,949	0	0.13	ts	132	AAG to AGG	Lys→Arg
	114	F	49	Mod	33	IB	6,998	0	0.1	ts	213	CGA to TGA	Arg→Stop
0<CS<25	55	F	68	Mod	42	IIIB	0	3.8	0.17	ts	242	TGC to TAC	Cys→Tyr
AB=0	126	M	66	Mod	30	IA	0	8	NA	ts	237	ATG to ATA	Met→Ile
≥25 CS	2	M	73	P	53	IIIB	0	39.4	0.25	ts	259	GAC to AAC	Asp→Ile
AB=0	12	M	69	Mod	28	IA	0	42.3	0.04	del	113–119	Del of 19 bp	Frameshift
	21	M	47	P	39	IIIA	0	32.5	0	tv	245	GGC to TGC	Gly→Cys
	42	M	58	P	24	IIIA	0	80	0.36	ts	273	CGT to TGT	Asp→Cys
	46	M	56	Mod	20	IIIB	0	31	0.1	del	159	GCC to---C	Frameshift
	54	M	54	Mod	25	IIIA	0	48	0.38	tv	198	GAA to TAA	Glu→Stop
	56	M	74	W	17	IA	0	42	0.24	ts	175	CGC to CAC	Arg→His
	58	M	61	P	23	IV	0	80	0.33	tv	135	TGC to TTC	Cys→Phe
	83	M	72	Mod	75	IV	0	126	0.44	del	274	GTT to---T	Frameshift
	88	M	50	Mod	48	IV	0	115.5	0.2	del	189	GCC to G---C	Frameshift
	96	M	54	Mod	27	IA	0	34	0.25	tv	158	CGC to CTC	Arg→Leu
	102	M	56	W	16	IA	0	37.5	0.06	ts	273	CGT to TGT	Asp→Cys
	116	M	50	P	60	IIIA	0	32	NA	tv	245	GGC to TGC	Gly→Cys
0<CS<25	17	M	58	W	27	IA	560	1.3	0.13	ts	234	TAC to TGC	Tyr→Cys
0<AB<1,000	128	F	69	P	60	IB	980	20	NA	ts	245	GGC to GAC	Gly→Asp
≥25 CS	11	M	64	Mod	20	IA	333	33	0.17	tv	Donor	AGgt to AGtt	Splicing
0<AB<1,000	49	M	41	P	105	IB	446	37.5	0.22	tv	Acceptor	agG to atG	Splicing
	97	M	72	Mod	16	IIIB	929	25.5	0.26	ts	273	CGT to CAT	Asp→His
	131	M	51	P	28	IIIA	339	31	NA	tv	244	GGC to TGC	Gly→Cys
0<CS<25	23	M	59	Mod	24	IA	1,538	24	0.12	tv	274	GTT to TTT	Val→Phe
≥1,000 AB	64	F	74	W	37	IIIB	1,477	12	0.11	tv	209	AGA to TGA	Arg→Stop
	86	M	49	Mod	23	IIA	2,039	1	0.45	tv	238	TGT to AGT	Cys→Ser
≥25 CS	16	M	72	P	35	IIIA	2,490	40	0.31	ts	158	CGC to CAC	Arg→His
≥1,000 AB	53	M	60	P	28	IIA	1,750	40	0.64	ts	158	CGC to CAC	Arg→His
	85	M	65	Mod	28	IA	2,337	45	0.55	ts	275	TGT to TAT	Cys→Tyr
	103	M	67	Mod	28	IIIB	1,293	30.6	0.2	ins	305–306	Ins of 23 bp	Frameshift
	104	M	74	Mod	32	IB	2,378	53	0.05	ts	Donor	AGgt to AGat	Splicing
	105	M	50	Mod	24	IA	2,212	58	0.29	del	179–185	Del of 18 bp	Frameshift
	109	M	47	P	64	IIIA	3,207	81	0.64	tv	158	CGC to CCC	Arg→Pro
	110	M	55	Mod	15	IA	3,881	35	0	ins	46	Ins of 16 bp	Frameshift
	115	M	71	Mod	20	IIIA	5,308	48	0.46	tv	157	GTC to TTC	Val→Phe

CS, cumulative smoking in pack-years; AB, asbestos burden; Diff., differentiation; pStage, pathological stage, FAL, fractional allelic loss; Mut, mutation; F, female; M, Male; Mod, moderately; W, well; P, poorly; ts, transition; tv, transversion; del, deletion; ins, insertion; NA, not analyzed. Specific codons in *p53* mutations characteristic of smoking are underlined.

**Table IV:** *p53* mutational spectra in lung adenocarcinomas, classified by AB and CS in pack-years.

			Transition									
							Transversion					
			CpG	Non-CpG								
Classified by CS and AB	No.	With *p53* mutation (%)	G:C to A:T	G:C to A:T	A:T to G:C	Total (%)	G:C to T:A	G:C to C:G	A:T to T:A	A:T to C:G	Total (%)	Del/Ins (%)
All cases	123	47 (38)	13	6	3	22 (47)	13	2	2	0	17 (36)	8 (17)
CS=0	49	14 (28)	6	1	2	9 (64)	3	1	0	0	4 (29)	1 (7)
AB=0	22	4 (18)	2	0	1	3 (75)	1	0	0	0	1 (25)	0 (0)
0<AB<1,000	13	4 (31)	2	0	0	2 (50)	2	0	0	0	2 (50)	0 (0)
≥1,000 AB	14	6 (43)	2	1	1	4 (67)	0	1	0	0	1 (17)	1 (17)
0<CS<25	17	7 (41)	1	2	1	4 (57)	1	0	2	0	3 (43)	0 (0)
AB=0	6	2 (33)	0	2	0	2 (100)	0	0	0	0	0 (0)	0 (0)
0<AB<1,000	3	2 (67)	1	0	1	2 (100)	0	0	0	0	0 (0)	0 (0)
≥1,000 AB	8	3 (38)	0	0	0	0 (0)	1	0	2	0	3 (100)	0 (0)
≥25 CS	57	26 (46)	6	3	0	9 (35)	9	1	0	0	10 (38)	7 (27)
AB=0	33	13 (39)	3	1	0	4 (31)	5	0	0	0	5 (38)	4 (31)
0<AB<1,000	7	4 (57)	1	0	0	1 (25)	3	0	0	0	3 (75)	0 (0)
≥1,000 AB	17	9 (53)	2	2	0	4 (44)	1	1	0	0	2 (22)	3 (33)

Percentages may not total 100, due to rounding. Del/Ins, deletion/insertion; AB, asbestos burden; CS, cumulative smoking.
